# Potential value of expression of receptor accessory protein 4 for evaluating the prognosis of lower-grade glioma patients

**DOI:** 10.18632/aging.205695

**Published:** 2024-03-28

**Authors:** Shuping Luo, Zhendong Liu, Haigang Chang, Xingbo Cheng, Rongjun Qian, Yanzheng Gao, Chaofeng Hou

**Affiliations:** 1 Department of Colorectal Surgery, Zhengzhou Central Hospital Affiliated to Zhengzhou University, Zhengzhou, Henan, China; 2 Department of Surgery of Spine and Spinal Cord, Henan Provincial People’s Hospital, People’s Hospital of Zhengzhou University, People’s Hospital of Henan University, Zhengzhou 450003, Henan, China; 3 Department of Neurosurgery, The First Affiliated Hospital of Xinxiang Medical University, Weihui, Henan, China; 4 Department of Neurosurgery, Henan Provincial People’s Hospital, People’s Hospital of Henan University, People’s Hospital of Zhengzhou University, Zhengzhou 450003, Henan, China

**Keywords:** REEP4, lower-grade glioma, prognosis, immunotherapy, DNA methylation

## Abstract

Background: REEP4 is involved in the regulation of the biological process of mitosis. Lower grade glioma (LGG), as a malignant tumor, is accompanied by abnormalities in mitosis, but there have been no reports of REEP4 so far.

Methods: We collected transcriptome data, DNA methylation data and the clinical characteristics of thousands of patients with LGG. Various big data analysis methods and molecular biology experiments were employed to reveal the impact of REEP4 on the pathological process of LGG.

Results: It was found that the expression of REEP4 was significantly elevated and negatively regulated by its methylation site. Therefore, both the high expression of REEP4 and low methylation state of cg16311504 showed that the patients are correlated with lower patient survival rate. In addition, high REEP4 expression participates in the regulation of various cancer-related cellular signaling pathways, such as the cell cycle, MAPK signaling pathway, NOD-like receptor signaling pathway, etc. More importantly, the level of immune cell infiltration significantly increased in the high expression group of REEP4 in the LGG tumor microenvironment and REEP4 has a high positive correlation with PD-L1 and other immune checkpoints.

Conclusions: In brief, this study is the first to introduce REEP4 in malignant tumors, which can be used as an independent risk factor that participates in the malignant process of LGG. More importantly, REEP4 has the potential to become a new star in the field of anti-tumor treatment.

## INTRODUCTION

Gliomas account for 80% of central nervous system malignancies and have high mortality and disability rates [1]. The TCGA database considers the World Health Organization (WHO) grading system, grade II and III gliomas as lower grade gliomas (LGGs). Although LGG has lower invasiveness compared to glioblastoma, its cellular heterogeneity makes it difficult for clinical diagnosis and treatment to eradicate. A total tumor resection and adjuvant radiotherapy and chemotherapy treatment scheme system has been established with the development of intraoperative imaging and biology; however, the five-year survival rate of LGG patients remains unsatisfactory [2]. The primary factor leading to this treatment dilemma is the insufficient understanding of the pathogenesis and tumor microenvironment of glioma; thus, setting up highly specific and sensitive treatment measures is impossible [3].

The target discovery of anti-tumor immunotherapy is crucial for the research and development of anti-tumor drugs [4]. With the continuous improvement of high-throughput sequencing technology and bioinformatics methods, anti-tumor immunotherapy targets with high sensitivity and specificity have been identified and were gradually confirmed using clinical trials [5]. For example, cytotoxic T lymphocyte antigen 4 (CTLA4) plays an important regulatory role in the functional state of the regulatory T-cell; thus, it is involved in the formation of the tumor suppressive immune microenvironment. Anti-tumor immune drugs (ipilimumab) targeting CTLA4 can, therefore, markedly improve the prognosis of patients with advanced tumors [6, 7]. Besides, PD-L1 is also an immune checkpoint for anti-tumor therapy. Because it is expressed by immunocytes, blocking its transmission pathway can improve the ability of immune cells to kill tumor cells so as to exert its anti-tumor effects [8, 9]. However, the toxic and side effects of immune checkpoint inhibitors (ICIs) are inevitable in clinical experiments. For example, ICIs can not only cause some autoimmune diseases, but also cause significant damage to the thyroid and pituitary glands, as well as other organs [10]. The low complete response rate of both PD-1 and CTLA4 to antitumor immunotherapy for glioma must still be emphasized [6, 11]. Therefore, finding new targets for immunotherapy and reducing toxic and side effects is crucial.

The uncontrolled proliferation of tumor cells, which is closely related to cellular mitosis, is one of the basic characteristics of tumor cells [12]. REEP4, located in the high curvature membrane of the cytoplasmic endoplasmic reticulum, plays an important regulatory role in the formation of nuclear pore complexes (NPCs) in the late stages of mitosis, [13] which led us to consider whether REEP4 may be involved in the promotion of tumor cell proliferation in the pathological tumor process. NPCs, as they are nucleocytoplastic transporters, are significantly increased in a variety of tumors. However, reducing their formation can inhibit the proliferation ability of tumor cells and induce cell death [14]. Subsequent searches revealed no evidence that REEP4 was related to any tumor; however, it was found that REEP3, which is in the same family as REEP4, was significantly increased in the malignant process of hepatocellular carcinoma and promoted tumor cell proliferation [15]. Therefore, we speculate that REEP4 may participate in the pathological evolution of gliomas and play an important regulatory role.

To explore whether REEP4 is indeed involved in the pathological process of glioma, we chose lower-grade glioma (LGG) as the object of study due to its great heterogeneity with GBM [16]. To our knowledge, this study systematically revealed, for the first time, that High levels of REEP4 are correlated with a significant reduction in survival time of patients with LGG. This can be used as a highly sensitive biological target for diagnosing and treating LGG, and makes up for the lack of focus on REEP4 in the study of tumor pathogenesis. More importantly, it systematically revealed the regulatory relationship between REEP4 and immune cell infiltration level in tumor microenvironment, especially M2 tumor related macrophages and the exploration of the potential value of anti-tumor immunotherapy.

## RESULTS

### Abnormal increase of REEP4 expression in LGG pathological process

**Figure 1:**
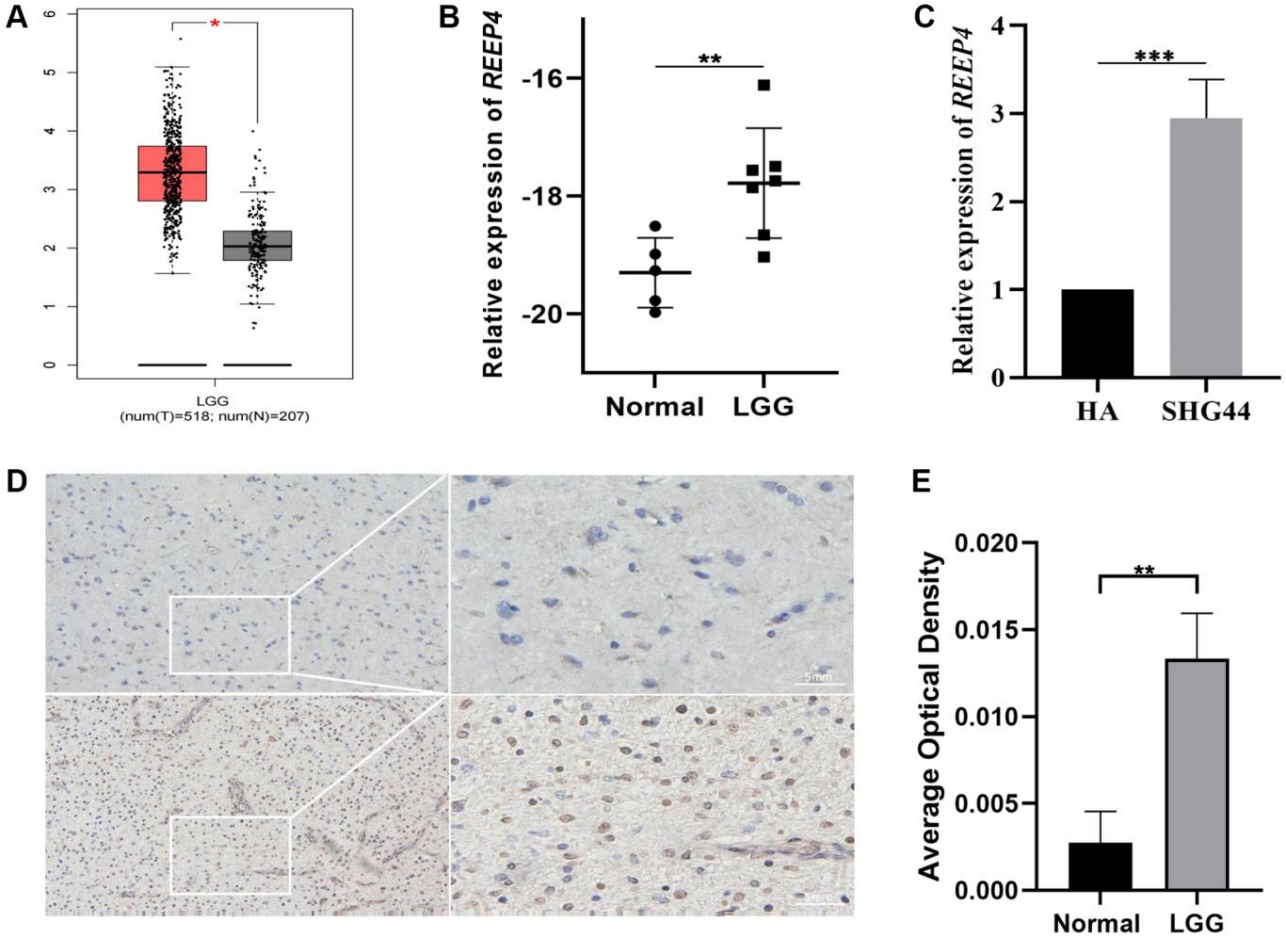
**Differential expression level of receptor accessory protein 4 (*REEP4*) in LGG and normal brain tissue.** (**A**) *REEP4* is significantly upregulated in 518 lower-grade glioma (LGG) and 207 normal brain tissues based on GEPIA. ^*^p < 0·05. (**B**) Real-time quantitative polymerase chain reaction (RT-qPCR) of clinical samples showing elevated *REEP4* expression in LGG tissues. ^**^p < 0·01. (**C**) RT-qPCR showing elevated *REEP4* expression in LGG cell lines. ^***^p < 0·001. (**D**, **E**) Immunohistochemistry shows elevated *REEP4* expression in LGG tissue compared with than that in normal brain tissue. ^**^p < 0·01.

Abnormally high expression of pathogenic genes is a common phenotype in the pathological progression of malignant tumors [17]. Therefore, to explore whether REEP4 is also abnormally overexpressed in the pathological process of LGG, we first searched the mRNA expression level of REEP4 in LGG in the GEPIA database based on unpaired *t*-test results. Our findings suggested that REEP4 expression is significantly higher in LGG than in normal brain tissue (Figure 1A). In addition, RT-qPCR revealed that the expression level of REEP4 in clinical tissue samples and LGG cell lines was significantly higher than that in the control group (Figure 1B, 1C). Finally, the translation of mRNA into protein is an important form of its regulatory effect. Therefore, we found that the protein expression level of REEP4 in LGG tissue samples was also significantly higher than that in the control group using immunohistochemical methods (Figure 1D, 1E). Collectively, these results suggest that REEP4 may play an important regulatory role in the malignant evolution of LGG.

### REEP4 has a positive expression relationship with the malignant clinical phenotype of LGG patients

**Figure 2:**
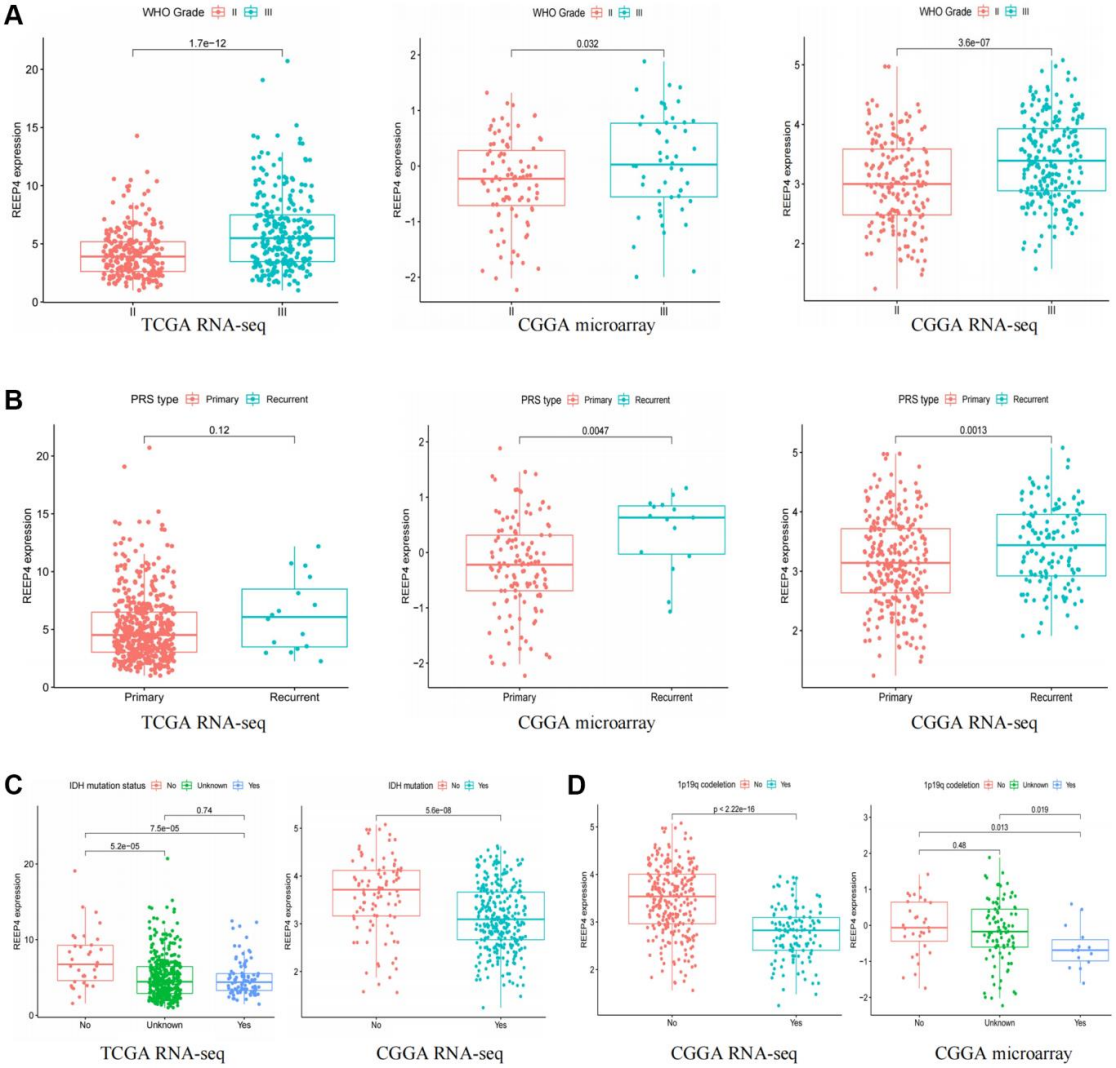
**Relationship between receptor accessory protein 4 expression, clinical features, and molecular typing.** (**A**) World Health Organization grade. (**B**) Primary, Recurrent, Secondary (PRS) type. (**C**) Isocitrate dehydrogenase mutation status. (**D**) 1p19q co-deletion status.

As the prognosis of patients with LGG is significantly correlated with their clinical characteristics [18], this study attempts to reveal the relationship between REEP4 expression and the clinical phenotype of patients with LGG based on the results of CGGA microarray, CGGA RNA-seq, and TCGA RNA-seq. The mRNA expression level of REEP4 was significantly higher in patients with WHO grade III and recurrence than in those with WHO grade II and primary disease (Figure 2A, 2B). Besides, the mRNA expression level of REEP4 was significantly higher in patients with wild type isocitrate dehydrogenase (IDH) and 1p19q non-codeletion than in IDH-mutation-type and 1p19q-co-deletion-type patients (Figure 2C, 2D). Furthermore, the mRNA expression level of REEP4 was significantly higher in chemotherapy and radiotherapy groups than in non-chemotherapy and non-radiotherapy groups (Supplementary Figure 1A, 1B). The mRNA expression level of REEP4 is higher in mesenchymal types than in classical, proneural, and neural types (Supplementary Figure 1C). Finally, the mRNA expression level of REEP4 also differed significantly among groups classified according to histological type (Supplementary Figure 1D–1F). Collectively, these results suggest that the high REEP4 expression may be closely related to the poor prognosis of LGG.

### Overexpression of REEP4 may be an independent risk factor for LGG prognosis

**Figure 3:**
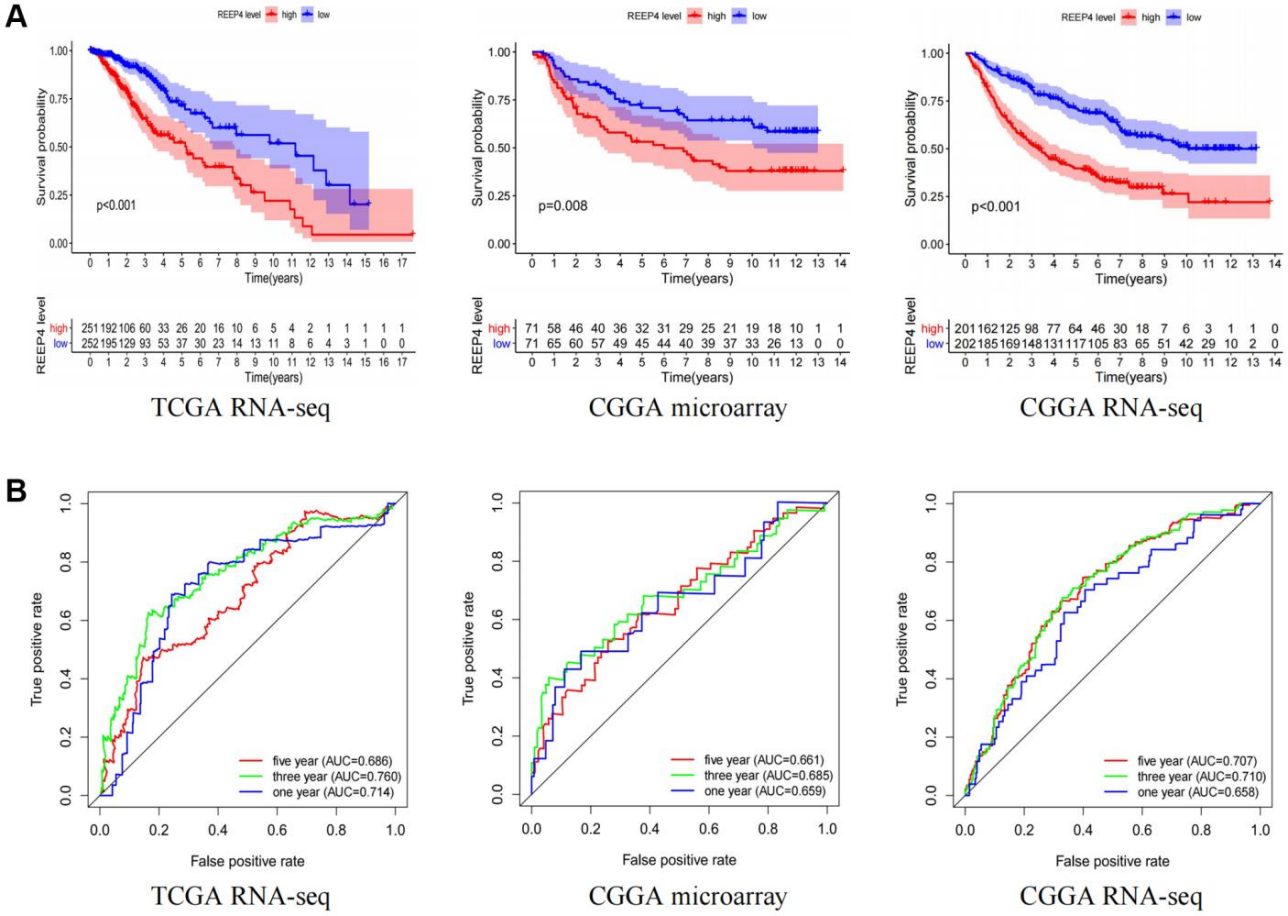
**The impact of REEP4 on the prognosis and diagnostic value of LGG patients.** (**A**) Kaplan-Meier analysis based on the different expression levels of receptor accessory protein 4 (REEP4) in lower-grade glioma (LGG). (**B**) Diagnostic value of *REEP4* in LGG by receiver operating characteristics.

It is significant that pathogenic genes lead to reduced survival time in patients with cancer [19]. Therefore, we tried to reveal the impact of REEP4 on the survival time of patients with LGG. Firstly, the results of Kaplan-Meier curves showed that the overall survival time of patients with LGG in the high REEP4 expression group was significantly lower than that in the low REEP4 expression group based on the CGGA microarray, CGGA RNA-seq, and TCGA RNA-seq (Figure 3A). It is worth emphasizing that the impact of REEP4 on the disease free survival time of patients with LGG has similar results (Supplementary Figure 2A). Subsequently, ROC analysis showed that high REEP4 expression had good diagnostic value for the prognosis of patients with LGG based on the above three datasets (Figure 3B). These results suggest that the high expression of REEP4 was associated poor prognosis of LGG.

To verify the above points, we first performed a meta-analysis to collect 1100 LGG patient samples from five different datasets. Each of the five independent datasets suggested that REEP4 was a risk factor for the overall survival of LGG, and the overall risk coefficient also suggested that REEP4 was a significant risk factor for the overall survival of LGG (Supplementary Figure 2B). The more important finding is that both univariate and multivariate analysis suggest that REEP4 can be used as an independent risk factor to affect the prognosis of patients with LGG (Supplementary Figure 2C–2H). In this part, various statistical methods and data sources are used to confirm that the high expression of REEP4 can be used as an independent risk factor as an ideal target for the diagnosis and treatment of LGG.

### The high expression of REEP4 is negatively regulated by its methylation in LGG

**Figure 4:**
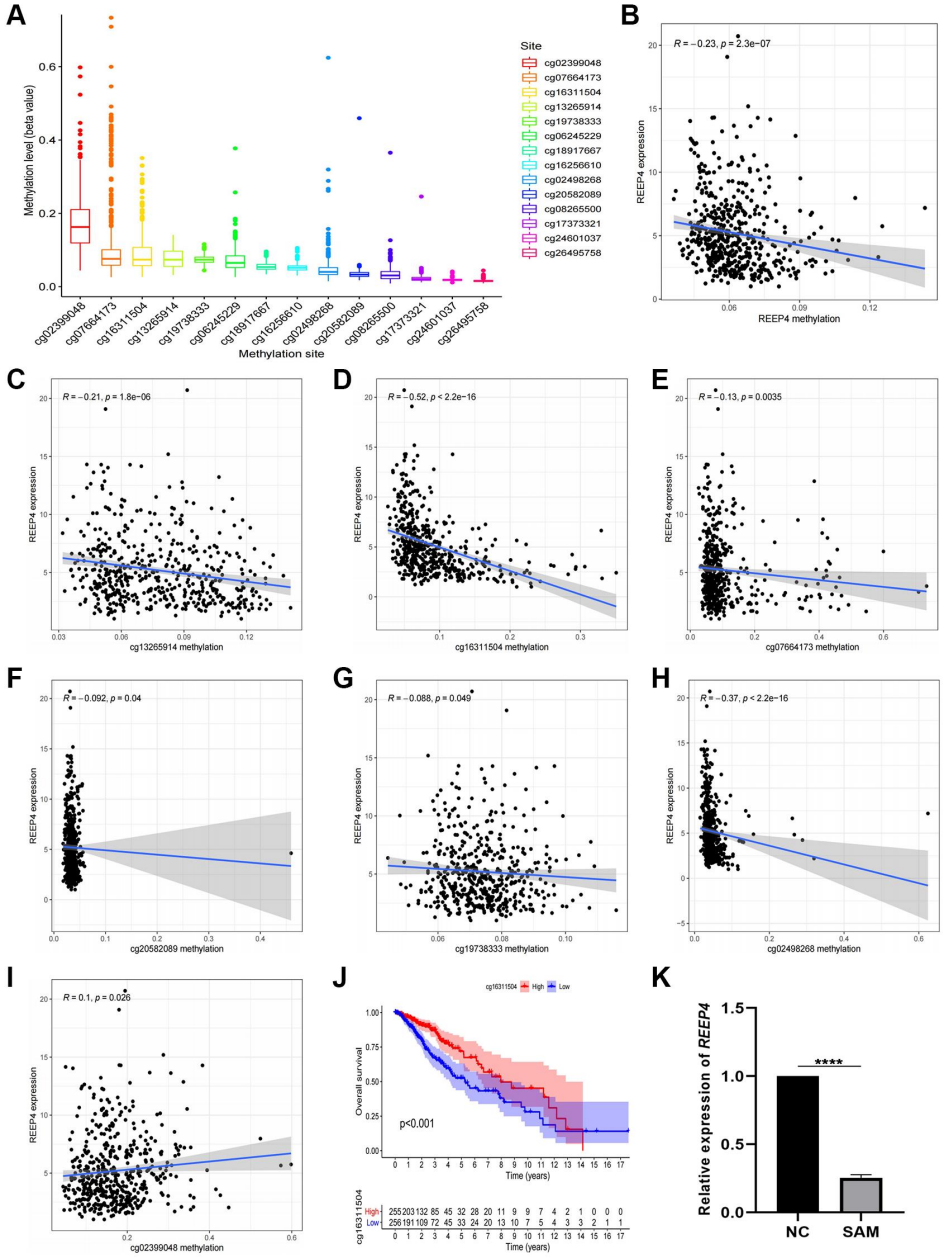
**Methylation level of receptor accessory protein 4 (*REEP4*) in lower-grade glioma (LGG) based on the TCGA database.** (**A**) Methylation sites of *REEP4* in LGG. (**B**) The expression level of *REEP4* is negatively correlated with the total methylation level. (**C**–**H**) The expression level of *REEP4* is negatively correlated with methylation sites cg13265914, cg16311504, cg07664173, cg20582089, cg19738333 and cg02498268. (**I**) The expression level of *REEP4* is positively correlated with the methylation site cg02399048. (**J**) Effect of methylation site cg16311504 on the overall survival of patients with LGG. (**K**) Real-time quantitative polymerase chain reaction showing that the expression level of *REEP4* was significantly inhibited by SAM. ^****^P < 0·0001.

Based on the effect of high expression of REEP4 on the prognosis of LGG, we aimed to reveal the reason for the increased expression of REEP4 in LGG. DNA methylation is an important epigenetic regulatory mechanism that has an important regulatory effect on mRNA expression [20]. Therefore, we analyzed the TCGA-RNA-seq and TCGA-DNA methylation data of REEP4 in LGG to reveal their relationship. First, we extracted the methylation status of 14 DNA methylation sites that regulate REEP4 expression and summarized them in Figure 4A. Second, co-expression analysis showed that the overall methylation level of the 14 DNA methylation sites exhibited a significant negative correlation with the mRNA expression level of REEP4 (Figure 4B). Third, co-expression analysis of single methylation sites showed that 6 of the 14 methylation sites (cg13265914, cg16311504, cg07664173, cg20582089, cg19738333, cg02498268) showed significant negative correlations with REEP4 expression (Figure 4C–4H). Only one methylation site (cg02399048) showed positive correlation (Figure 4I), and the remaining methylation sites showed no regulatory relationships. Fourth, the Kaplan-Meier curves showed that only one methylation site (cg16311504) affects LGG prognosis, and its hypermethylation status can prolong the overall survival of patients (Figure 4J). Meanwhile, clinical correlation analysis showed that the hypermethylation status of cg16311504 was negatively related to the clinical and molecular features associated with poor prognosis of patients with LGG (Supplementary Figure 3). This suggests that cg16311504 has a protective effect on LGG prognosis. Fifth, after the SHG-44 of LGG cell line was treated with SAM (methylating drug), RT-qPCR results revealed that REEP4 expression was significantly inhibited. Collectively, the hypothesis that the increased expression of REEP4 in the malignant process of LGG was negatively regulated by its methylation sites was confirmed.

### Co-expression analysis and enrichment analysis of KEGG cell signal pathway of REEP4 in LGG

**Figure 5:**
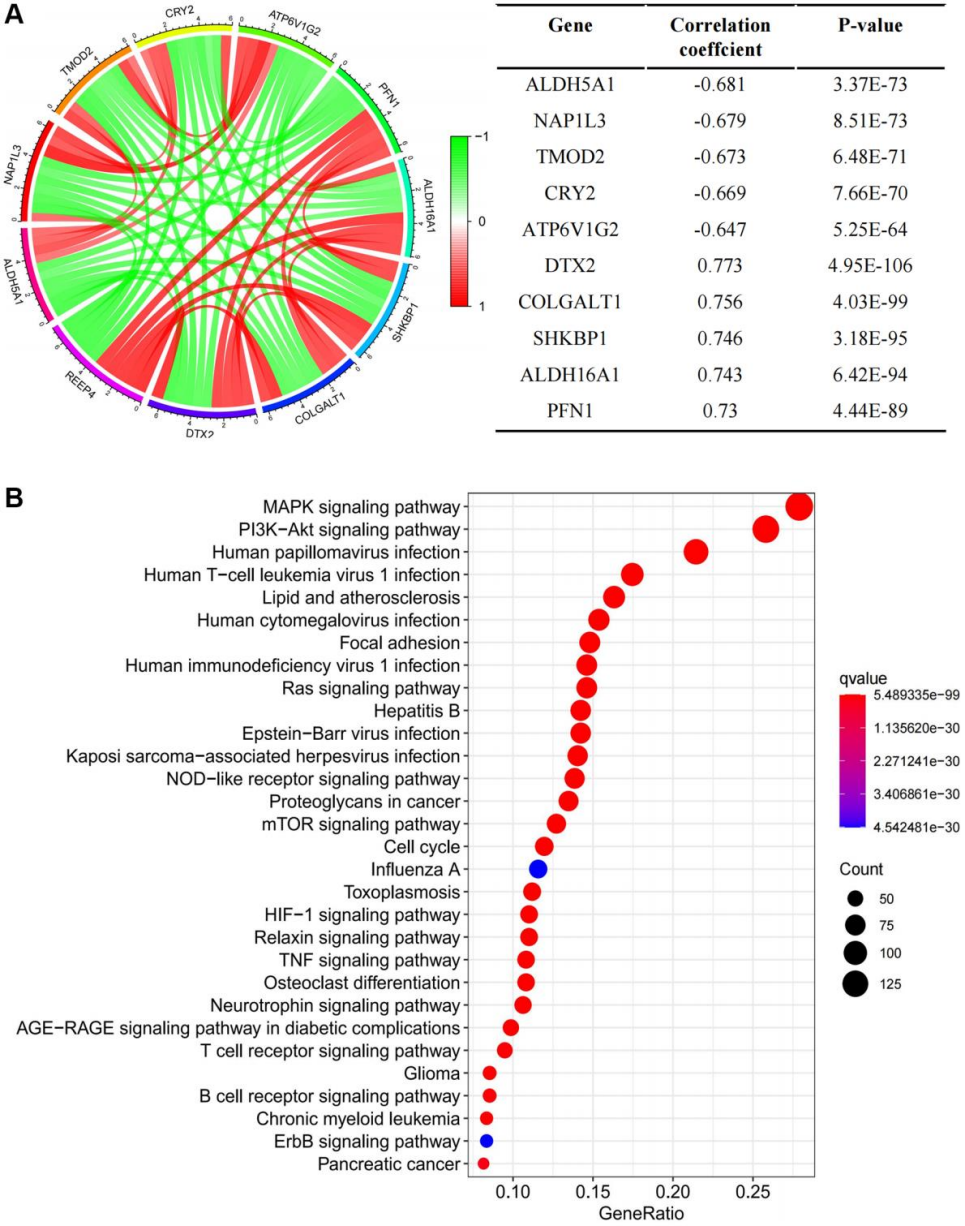
**Exploring the mechanism of REEP4 in the pathological progression of LGG.** (**A**) Gene co-expression analysis enumerating the first five genes that were positively and negatively correlated with receptor accessory protein 4 (*REEP4*) in lower-grade glioma (LGG). (**B**) KEGG signal pathway analysis showed the pathway of significant enrichment of *REEP4* in LGG.

To address how highly expressed *REEP4* participates in the regulation of LGG pathological progression, we first determined that *REEP4* was co-expressed with hundreds of genes through gene overexpression analysis and presented the top five genes with the most positive and negative correlations in Figure 5A. In addition, we found that REEP4 was primarily enriched in cancer-related cell signal pathways such as the cell cycle, MAPK signaling pathway, HIF-1 signaling pathway, NOD-like receptor signaling pathway, TNF signaling pathway, etc., (Figure 5B) through KEGG signal pathway analysis. Therefore, highly expressed REEP4 may play a regulatory role through the above signaling pathways in promoting the malignant progression of LGG. At the same time, further literature searches found that activating these cellular pathways not only promoted the proliferation and migration of tumor cells, but also played an important role in regulating tumor related immune cells.

### Highly expressed REEP4 participates in the formation of LGG inhibitory immune microenvironment

The tumor-specific immunosuppressive tumor microenvironment is closely related to the abnormal expression of transcriptomics [21]. Based on the above results, we tried to reveal whether abnormal REEP4 expression is involved in the formation of the LGG immune microenvironment. Therefore, using the TIMER database, we first found that REEP4 was negatively correlated with the purification of cancer cells and exhibited a high positive correlation with the degree of infiltration of five different immune cells (B cell, CD4+ T cell, macrophage, neutrophil, and dendritic cell) (Supplementary Figure 4A). Further survival analysis showed that the overall survival of patients with LGG in the high infiltration group with six infiltrating immune cells was significantly reduced (Supplementary Figure 4B). In addition, it is interesting that the copy number variation of REEP4 can also affect the degree of immune cell infiltration (Supplementary Figure 4C). These findings suggest that the adverse effect of REEP4 on the prognosis of patients with LGG may be partly induced by effects on the immune microenvironment.

**Figure 6:**
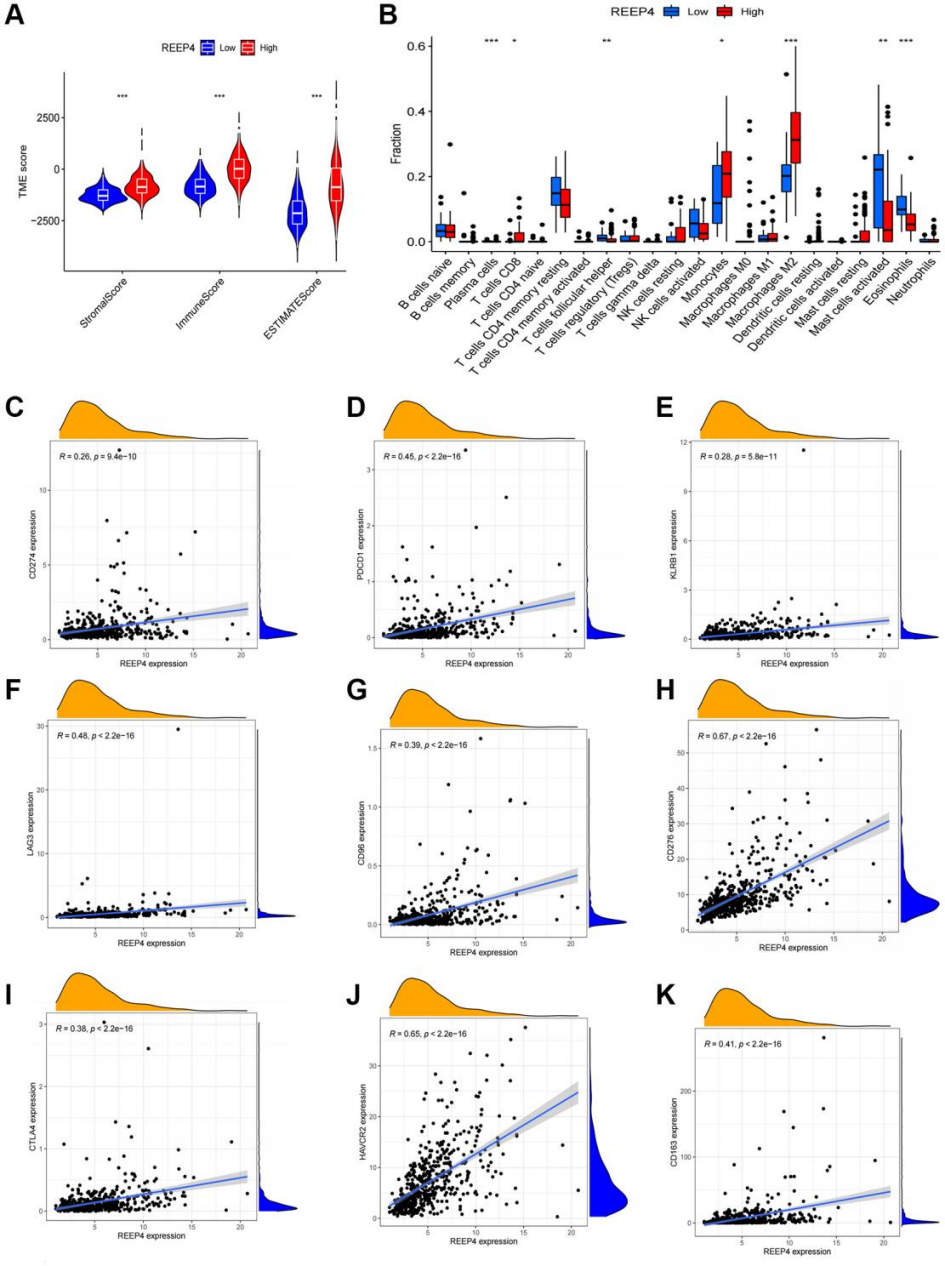
**The effect of REEP4 on the immune microenvironment of LGG.** (**A**) The expression level of receptor accessory protein 4 (*REEP4)* was positively correlated with the stromal, immune, and ESTIMATE scores. ^***^P < 0·001. (**B**) The correlation between *REEP4* and 22 immune cell subtypes. ^*^P < 0·05. ^**^P < 0·01. ^***^P < 0·001. (**C**–**K**) *REEP4* expression is positively correlated with 8 immune checkpoints, including CD274, PDCD1, KLRB1, LAG3, CD96, CD276, CTLA4, HAVCR2, CD163.

Due to the lack of subtype classification of immune cells in the TIMER database, we further used ESTIMATE and CIBERSORT to supplement and verify the impact of REEP4 on immune cell microenvironment based on TCGA-RNA-seq results. We first performed three groups of immune classification grouping (stromal score, immune score, and ESTIMATE score) and scoring, and found that the high REEP4 expression had higher scores than the low expression group (Figure 6A). In further immune cell typing, the expression level of REEP4 differed significantly among various immune cells, especially in M2 macrophages (Figure 6B). Subsequently, we focused on the relationship between REEP4 expression and the markers of 16 immune cells, and found that all immune cell markers were positively correlated with REEP4 in LGG except NOS2 (M1 Macrophage), which had a negative correlation (Table 1).

**Table 1:** Correlation between the expression of REEP4 and markers of immune cells based on TCGA-RNA seq database.

**Immune cell**	**Gene**	**Correlation coefficient**	***P*-value**
T cell (general)	CD3D	0.42130979	*P* < 0.001
	CD3E	0.420699315	*P* < 0.001
	CD2	0.435613013	*P* < 0.001
CD8+ T cell	CD8A	0.038063419	*P* = 0.382
	CD8B	0.298162186	*P* < 0.001
CD4+ T cell	CD4	0.618150853	*P* < 0.001
Monocyte	CD86	0.580877055	*P* < 0.001
B cell	CD19	0.557037403	*P* < 0.001
	CD79A	0.387402848	*P* < 0.001
TAM	CCL2	0.370916678	*P* < 0.001
	CD68	0.635256333	*P* < 0.001
	IL10	0.510947916	*P* < 0.001
M1 MacroPhage	NOS2	−0.185936846	*P* < 0.001
	PTGS2	−0.054298297	*P* = 0.212
M2 MacroPhage	CD163	0.414394534	*P* < 0.001
	VSIG4	0.499414167	*P* < 0.001
	MS4A4A	0.531418548	*P* < 0.001
NeutroPhil	ITGAM	0.555851197	*P* < 0.001
	CCR7	0.246631662	*P* < 0.001
Th1	TBX21	0.416241369	*P* < 0.001
	STAT1	0.246778607	*P* < 0.001
	TNF	0.173812284	*P* < 0.001
Th2	GATA3	0.480185131	*P* < 0.001
	STAT6	0.289126139	*P* < 0.001
	STAT5A	0.649674699	*P* < 0.001
	IL13	0.239178304	*P* < 0.001
Tfh	BCL6	0.166343176	*P* < 0.001
Th17	STAT3	0.388685487	*P* < 0.001
Treg	STAT5B	−0.067883916	*P* = 0.118
	FOXP3	−0.064351571	*P* = 0.139
	TGFB1	0.725450217	*P* < 0.001
T cell exhaustion	PDCD1	0.448847591	*P* < 0.001
	CTLA4	0.379607713	*P* < 0.001
	LAG3	0.478496678	*P* < 0.001
	HAVCR2	0.648187298	*P* < 0.001
	GZMB	0.202322014	*P* < 0.001
Dendritic cell	HLA-DPB1	0.597150762	*P* < 0.001
	HLA-DQB1	0.507724695	*P* < 0.001
	HLA-DRA	0.570111411	*P* < 0.001
	HLA-DPA1	0.528122984	*P* < 0.001
	CD1C	0.286001626	*P* < 0.001
	NRP1	0.206953069	*P* < 0.001
	ITGAX	0.646464331	*P* < 0.001

Finally, anti-tumor immunotherapy is a current research hotspot. Identifying new immune checkpoints will help researchers develop new drugs for anti-tumor immunotherapy [22]. Therefore, we tried to reveal the relationship between REEP4 expression and known immune checkpoints based on TCGA-RNA-seq results and found that REEP4 had a high positive expression relationship with the eight most common immune checkpoints (Figure 6C–6K). Therefore, revealing the relationship between REEP4 and immune checkpoints will help to improve the progress of anti-tumor immunotherapy.

## DISCUSSION

In the process of biology, REEP4 participates in the process of regulating cellular mitosis [13], thereby affecting the proliferation of tumor cells. Because LGG is a common malignant tumor in the brain, its cells have the characteristics of abnormal proliferation [23]. Therefore, we try to reveal whether REEP4 exerts a regulatory effect on the pathological process of LGG. First, we found that the mRNA expression level of REEP4 in LGG was significantly increased using the GEPIA database; thus, we speculated that REEP4 may be involved in regulating the pathological process of LGG. To further confirm the reliability of the analysis results, RT-qPCR and immunohistochemical methods were used to verify that the expression level of mRNA or protein REEP4 was indeed significantly increased in tissues and tumor cells. Lastly, previous studies have confirmed that abnormal gene expression in malignant tumors will affect patient prognosis [24]. For example, the abnormally high expression of HOTAIR in glioma can significantly reduce the survival time of patients and can be used as a target for diagnosis and treatment [25, 26]. Since REEP4 has not been reported in cancers to date, whether the abnormal high expression of REEP4 has an impact on the prognosis of LGG aroused a strong interest in this matter on our part.

One characteristic of oncogenes is that it can promote the malignant pathological process of tumors and adversely affect patient prognosis [27]. Therefore, we first reveal the relationship between REEP4 and the clinicopathological features of LGG patients through the chi-squared test because the prognosis of patients with LGG is significantly correlated with different clinical features that can be divided into LGG subtypes. The results show that the mRNA expression level of REEP4 was significantly higher in WHO grade III and recurrence than in WHO grade II and primary disease. Previous studies have shown that the patients with LGG in the recurrence group and WHO grade III will have a worse prognosis [28, 29]. Besides, the mRNA expression level of REEP4 was significantly higher in IDH-wildtype and 1p19q non-codeletion than in IDH-mutation-type and 1p19q-codeletion-type. However, the patients with LGG with IDH-mutation-type and 1p19q-co-deletion type have better prognoses [30]. The above arguments suggest that abnormally high REEP4 expression in LGG was associated poor prognosis of LGG.

Genes that play an important role in regulating the pathological process of tumors can often affect patients’ survival time [27]. Therefore, the study first used Kaplan-Meier curves and found that the increased expression of REEP4 was not only significantly associated the overall survival, but also the disease-free progression of patients with LGG. Based on this result, we speculate that REEP4 may be a key risk factor in the pathological process of LGG. We then used Cox’s method to verify this view, and three data sets from different sources suggest that REEP4 is an independent risk factor for the overall survival time of patients with LGG. Previous studies also support our view that elevated gene expression in tumor tissues can be identified as a pathogenic factor that can worsen patient prognosis [31]. For example, the significant increase in BCL7A during the pathological process of glioma can significantly reduce patients’ overall survival time, and has diagnostic value for the prognosis of patients [32]. In addition, TUBA1C, SBF2-AS1, and SMYD2 have similar expression and prognostic impact in glioma [33–35]. In conclusion, the present study confirmed that abnormally high expression of REEP4, as an independent risk factor was associated poor prognosis of LGG. It is further speculated that REEP4 may become a valuable biological target for further application in drug research and development.

So, why is REEP4 significantly higher in LGG at both transcriptome level and protein expression level? According to the theory of epigenetics, different states of DNA methylation can regulate downstream gene expression [36], which provides a theoretical basis for answering the question of the increased expression of REEP4 in LGG. Therefore, we speculate that elevated REEP4 expression is caused by a reduction in DNA methylation. To confirm this hypothesis, we first extracted 14 DNA methylation states that regulate REEP4 expression. Co-expression analysis then showed that the DNA methylation level of REEP4 indeed showed a significant negative regulatory relationship with its expression level. More importantly, we first used SAM (methylating drugs) to further verify the results and thereby improve the methylation level of the SHG-44 of LGG cell line [37]. We then used RT-qPCR to confirm that the expression level of REEP4 was indeed significantly reduced. Therefore, we confirmed that a reduction in DNA methylation led to increased REEP4 expression in the pathological process of LGG. However, it must be noted whether the abnormal state of the DNA methylation states of REEP4 will affect the survival time of LGG patients. To solve this problem, we used Kaplan-Meier curves and found that hypermethylation of only one methylation site (cg16311504) was significantly associated the survival time of patients with LGG. Therefore, we speculate that cg16311504 may be further used as a biomarker to improve the prognosis of patients with LGG.

Oncogenes can promote cancer through numerous biological processes in the pathological process of malignant tumors [38]. Therefore, the present study next attempts to reveal the regulatory mechanism of REEP4 in the pathological process of LGG. The characteristics of gene co-expression often have similar or antagonistic effects in the pathological process of disease. Therefore, we first used co-expression analysis to show that most genes with positive relationships with REEP4 are oncogenes, and most genes with a negative expression relationship with REEP4 are tumor suppressor genes (Figure 5A). This is in line with the above theoretical basis. For example, the CRY2, ALDH5A1, and ATP6V1G2 tumor suppressor genes are involved in osteosarcoma, ovarian cancer, and glioma [39–41], respectively. Additionally, the oncogenes, ALDH5A1 and NAP1L3, exerted effects in papillary thyroid carcinoma and hepatocellular carcinoma, respectively [42, 43]. Secondly, we used KEGG analysis to further reveal the effect of REEP4 on the cell signaling pathway in LGG. The results suggest that the cellular signaling pathway regulated by REEP4 is involved in the proliferation, differentiation, and immune microenvironment of cancers (Figure 5B). For example, the cell cycle and MAPK signaling pathway can significantly regulate the proliferation and differentiation of glioma cells [44]. In addition, the HIF-1, NOD-like receptor, and TNF signaling pathways exert significant regulatory effects on numerous immune cells [45–47]. Most immune cells are in a remodeling state in the LGG microenvironment [48], suggesting that REEP4 may also be involved in the regulation of the LGG immune microenvironment.

Based on the potential value of anti-tumor immunotherapy, this study further explored the impact of REEP4 on the LGG immune microenvironment. We first found that the high expression of REEP4 is significantly correlated with the high infiltration of six different immune cells (B cell, CD4+ T cell, macrophage, neutrophil, dendritic cell) in the TIMER database, which was significantly associated the survival time of patients with LGG. Previous reports have also shown that LGG is a tumor with high immune cell infiltration; thus, it may be useful in anti-tumor immunotherapy [49]. Among so many immune cells, this study primarily discusses the relationship between REEP4 and macrophages. Therefore, macrophages with different polarization states can have different effects on the prognosis of LGG [50]. Due to the lack of analysis functions for immune cell subtypes in the TIMER database, we used co-expression analysis, ESTIMATE, and CIBERSORT to find that REEP4 primarily promoted the infiltration level of M2 macrophages in LGG. However, M2 macrophages are the primary component of tumor-related macrophages, which can promote neovascularization and the proliferation of tumor cells, and also secrete a variety of cytokines, resulting in a significant reduction in the survival time of patients with LGG [51, 52]. To determine whether REEP4 was significantly associated the infiltration with high M2 macrophages, we further confirmed the positive relationship between the expression of REEP4 and CD163 in LGG samples. Subsequently, we considered whether REEP4 has become a target of anti-tumor immunotherapy. Co-expression analysis shows that REEP4 has a high positive expression relationship with various existing well-known immune checkpoints (Figure 6C–6K). Based on the above discussion, we speculate that the tumorigenic effect of REEP4 in LGG may be partially achieved by regulating immunity and that REEP4 may be a valuable new target for antitumor immunotherapy.

Although this study has comprehensively discussed the biological effects of REEP4 in the malignant progression of LGG, no article is absolutely flawless. The following are the shortcomings of this article. Firstly, the data in this study mainly comes from public databases and has the attribute of multiple central sources. Therefore, there may be inconsistencies in the collected standards between the data, which may lead to bias in the final results. Secondly, this study falls within the scope of retrospective research and therefore carries inherent defects, which cannot be avoided. Thirdly, this study revealed multiple biological effects of REEP4 in the pathological process of LGG but did not have the ability to validate all analysis results one by one, resulting in insufficient persuasiveness in this study. The above issues will be addressed in our future reports.

Taken together, through transcriptomic and DNA methylation data of LGG, we answered why the expression of REEP4 is increased in LGG and elucidated its impact on the prognosis of LGG patients, for the first time to our knowledge. Our findings suggested that REEP4 or its methylation site cg16311504 may become a biological target for the treatment of LGG. In addition, the impact of REEP4 on the tumor immune microenvironment and the potential value of anti-tumor immunotherapy were revealed for the first time, broadening the understanding of molecular biology of REEP4. However, the fly in the ointment is that this analysis involves too many aspects of mechanism analysis. A single article cannot comprehensively verify the results of the analysis.

## MATERIALS AND METHODS

### Data collection

Gene Expression Profiling Interactive Analysis (GEPIA: http://gepia.cancer-pku.cn/) is an online analysis data platform that integrates the transcriptome sequencing of tumor tissues in The Cancer Genome Atlas (TCGA) database and the normal tissue samples from the GTEX projects to explore the changes in expression and prognosis of target genes in tumor tissues [53]. In the present study, we used the GEPIA database to search the expression changes of REEP4 and its impact on disease-free survival in LGG.

TCGA database (https://portal.gdc.cancer.gov/) is a professional data platform for cancer research of individuals of Caucasian and African descent and includes various data entries on cancer patients, e.g., transcriptome sequencing, DNA methylation, somatic mutation, etc., [28]. In the present study, we collected 503 RNA-seq and 511 DNA methylation data entries and corresponding clinical and molecular characteristics of the patients. Additionally, detailed patient information has been included in Supplementary Table 1. The above data were downloaded through the Xena portal (https://xenabrowser.net/) and used to explore the correlation between REEP4 and the prognosis, clinical features, DNA methylation, and immune microenvironment of patients with LGG.

The CGGA database is a public data platform for the pathological mechanism of human gliomas of Asian descent [54]. In this database, we obtained two types of data on LGG, namely CGGA microarray (142 cases) and CGGA RNA-seq (403 cases), as well as the corresponding patient clinical information (Supplementary Tables 2 and 3). The above data were used to verify the prognostic impact of REEP4 on LGG in TCGA database.

In addition to the above public database, we also collected brain tissue samples from 5 patients with epilepsy and 7 with LGG, which were used to perform RT-qPCR to evaluate the change in REEP4 mRNA expression level. Additional tissue samples from 3 patients with epilepsy and 3 with LGG were used to detect the protein expression levels of REEP4 using immunohistochemistry. All tissue samples were obtained from the operating room of the Henan Provincial People’s Hospital and placed into a −80° refrigerator for storage until use. The collection of samples was approved by the Ethics Committee of Henan Provincial People’s Hospital (2020107).

### Cell culture and treatment

To validate the expression patterns of REEP4 at the cellular level, human astrocyte (HA) cell lines and the SHG-44 of LGG cell line were selected for *in vitro* experiments. Cells were cultured using a complete medium formulated with 89% high glucose medium (Cat PM150210, Procell, China), 10% fetal bovine serum (FBS) (Cat 10099141, Gibco, USA), and 1% penicillin and streptomycin (Cat P1400, Solarbio, China). They were then incubated in a constant temperature incubator at 37°C with 5% CO_2_. When cell fusion approached 100%, the cells were passaged at 50% and cultured for subsequent experiments. For the methylation assay, SHG-44 cells were treated with 100 uM of ademetionine disulfate tosylate (SAM) when their cell fusion approached 70% (Cat 97540-22-2, Topscience, China) for 10 h. The treated cells were harvested to identify the REEP4 RNA level.

### RNA isolation and RT-qPCR

Total RNA was collected in accordance following the instructions of Total RNA Kit I (R6834-02, Omega, USA) and the concentration thereof was measured using NanoDrop (Thermo Fisher Scientific, USA). The cDNA was synthesized on the T100 Thermal Cycler (Bio-Rad, USA) as per the manufacturer’s instructions of NovoScript Plus All-in-one 1st Strand cDNA Synthesis SuperMix (Novoprotein, China) and quantitatively analyzed to determine the expression of *REEP4* mRNA on StepOne Plus Real-Time PCR System equipment (Thermo Fisher Scientific) in accordance with the manufacturer’s instructions of NovoStart^®^ SYBR qPCR SuperMix Plus (Novoprotein). An internal reference gene, *18S*, was used to standardize the mRNA expression of *REEP4*. The primer sequences of *18S* and *REEP4* are as follows. The results of cell and tissue RT-qPCR were calculated using the log_2_^−ΔΔCT^ and ^−ΔCT^ modes, respectively. The forward and reverse primers of 18S are as follows: 5′-GTAACCCGTTGAACCCCATT-3′ and 5′-CCATCCAATCGGTAGTAGCG-3′, respectively. The forward and reverse primers of *REEP4* are as follows: 5′-TCGTGCTGTGGCTGCTCTCA-3′ and 5′-CGATCTCCTTCTCATGGCGG-3′, respectively.

### Immunohistochemistry staining

The frozen brain tissue samples were removed from the liquid nitrogen, and after being fixed, dehydrated, embedded, and sliced, the 4-μm-thick paraffin sections were used to detect the protein expression of *REEP4*. Firstly, after being dried in an oven at 55°C for 30 min, the sections were dewaxed in xylene twice for 10 min and dehydrated in 85% absolute ethanol, 95% absolute ethanol, 100% absolute ethanol and 100% absolute ethanol for 10 min one by one. The sections were then immersed in EDTA antigen repair solution (ZSGB-BIO, China) and heated in a microwave for 20 min, and the endogenous peroxidase was inactivated and 10% serum solution was used to block nonspecific antigen for 30 min. Lastly, the sections were incubated overnight in the 4°C refrigerator with the primary antibodies of *REEP4* (1:50, Proteintech, China), respectively, The next day, after the secondary antibody was incubated for 1 h and the chromogenic solution was used to develop color, the IHC staining results were photographed under the 200× microscope and calculated by ImagePro-Plus software (version 6.0).

### Meta-analysis of REEP4 in LGG

To confirm whether REEP4 is a risk factor for the prognosis of patients with LGG, more data were collected through meta-analysis to improve the reliability of the analysis results. Five datasets from different sources (CGGA microarray: 142 cases; CGGA RNA-seq: 403 cases; TCGA RNA-seq: 503 cases; GSE43378: 18 cases; GSE50025: 34 cases) were included in this analysis. The GSE43378 and GSE50025 datasets were obtained from GEO database, and the gene symbols were transformed based on GPL570 and GPL13938 respectively [55, 56]. The above data sets first used the Cox method to obtain the risk coefficient of REEP4 on the overall survival of patients with LGG in each independent dataset, then collected five different risk coefficients through meta-analysis using the “Meta” package in R software (Version number: R x64 4·0·3). Since I_2_ statistics of >50 existed among the five datasets, a random-effects model was selected to complete the meta-analysis.

### Difference analysis and KEGG enrichment analysis of REEP4

To reveal the effect of REEP4 on cell signaling pathway in LGG, we classified all samples into high and low expression groups according to the median expression level of REEP4 based on TCGA RNA-seq. Hundreds of differentially expressed genes between the two groups were then filtered out based on the criteria with a log fold change of >1 and *P*-value of < 0·05 [28]. The subsequent enrichment analysis of KEGG cell signal pathway was completed using the “clusterprofiler” package in R software based on a *q*-value of < 0·05. The “ggplot2” package in R software was used to graph meaningful results.

### Immune correlation analysis of REEP4 in LGG

The TIMER database (https://cistrome.shinyapps.io/timer/) is an online analysis platform for a series of algorithms to reveal the impact of target genes on tumor immune microenvironments [57]. Firstly, this study searched the TIMER database to determine the relationship between the expression or variation of REEP4 and six different degrees of immune cell infiltration, as well as the impact of high infiltration of immune cells on patient prognosis.

ESTIMATE is a method used to assess tumor purity by estimating the proportion of mesenchymal and immune cells in tumor samples using gene expression signatures [58]. To study the relationship between REEP4 and tumor purity, we divided LGG samples into two groups with high and low expression according to the median expression level of REEP4 based on TCGA RNA-seq. Afterwards, the differences in the stromal, immune, and estimated scores of the two groups of patients were compared using the ESTIMATE package in R software. To further explore the relationship between REEP4 and the LGG tumor microenvironment, we used CIBERSORT, the current most widely used immune cell infiltration algorithm tool, to conduct further research [59]. In this study, we used the CIBERSORT package of R software to evaluate differences in immune cell infiltration for each sample in two groups of samples with high and low REEP4 expression.

### Statistical analysis

The results of RT-qPCR and immunohistochemistry were statistically analyzed using the unpaired *t*-test. The chi-squared test, Pearson’s correlation coefficient, Kaplan-Meier curves, and Cox expression models were used to perform clinical correlation, co-expression, survival, and multivariate analyses, respectively. Time-dependent receiver operating characteristic (TD-ROC) curves were plotted to assess the diagnostic value of REEP4 in the prognosis of patients with LGG. All statistical results with *p*-values of < 0·05 are considered significant.

### Role of the funding source

Transcriptomic data and clinicopathological characteristics of LGG patients were obtained from the following datasets: CGGA microarray: 142 cases; CGGA RNA-seq: 403 cases; TCGA RNA-seq: 503 cases; GSE43378: 18 cases; GSE50025: 34 cases.

### Availability of data and materials

Most of the patient samples in the Bioinformation Analysis part are from various public databases, but the patient samples in the validation part are from the operating room of Henan Provincial People’s hospital.

## Supplementary Materials

Supplementary Figures

Supplementary Tables
