# Supplementary material for: Potential value of expression of receptor accessory protein 4 for evaluating the prognosis of lower-grade glioma patients
Source: Aging (Albany NY). 2024 Mar 28;16(7):6188–211. doi: 10.18632/aging.205695 (PMC11042925; doi:10.18632/aging.205695)
Supplement: Supplementary Figures [file aging-16-205695-s001.pdf]

SUPPLEMENTARY FIGURES

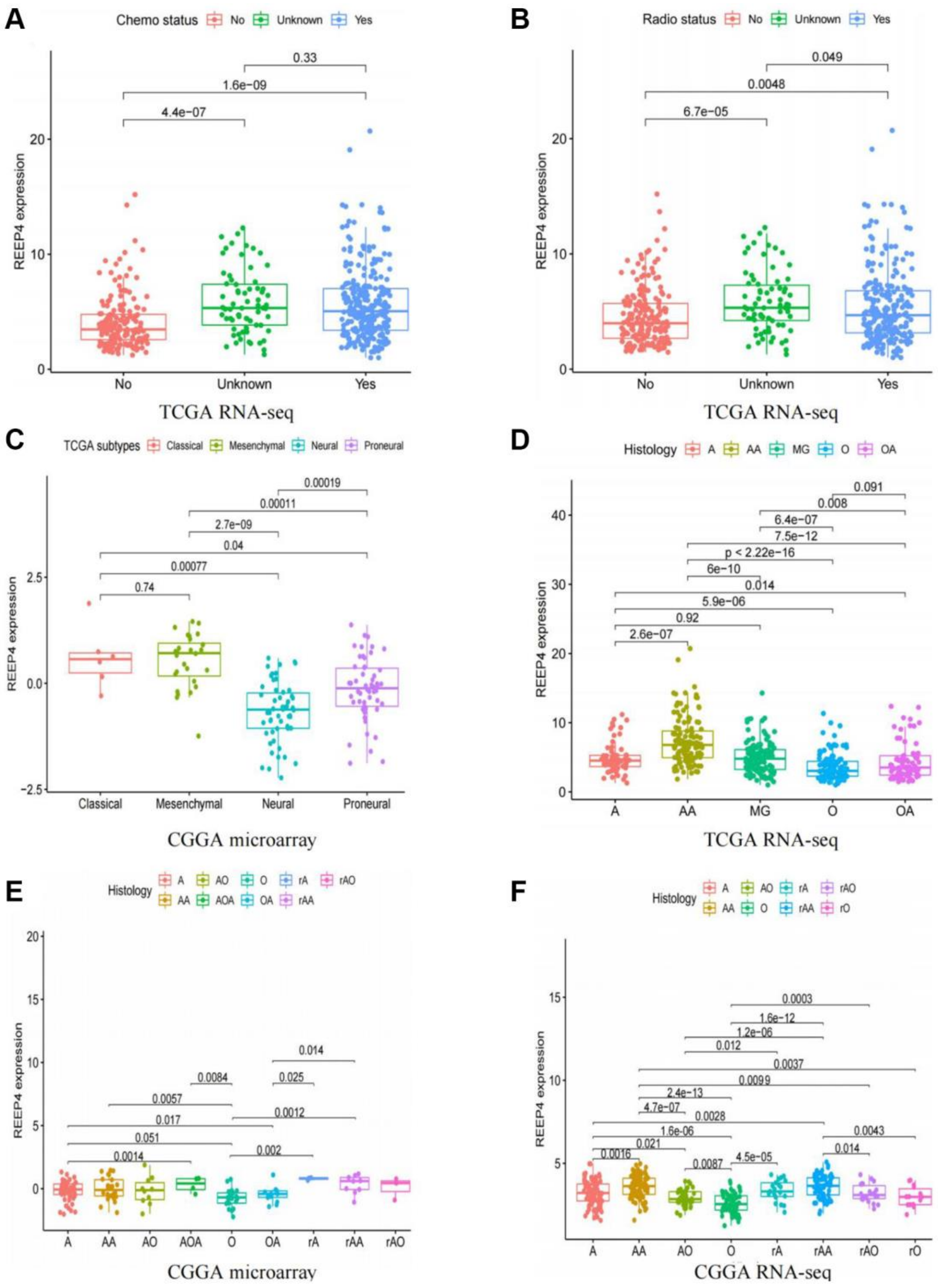

Supplementary Figure 1. Relationship between receptor accessory protein 4 (REEP4) expression and clinical features and histology type. (**A**) Chemotherapy status. (**B**) Radiotherapy status. (**C**) TCGA subtype. (**D**–**F**) Histological subtype.

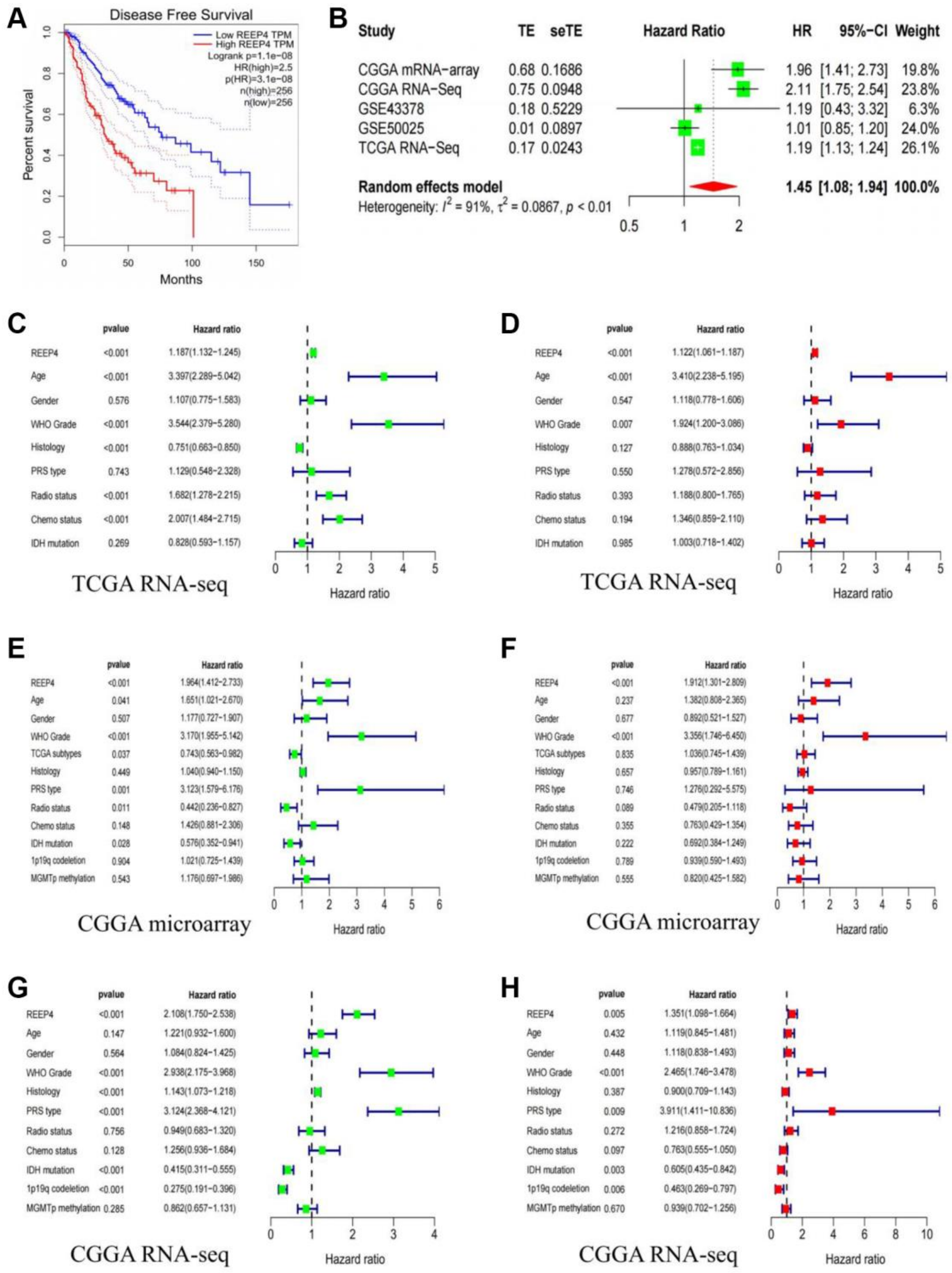

Supplementary Figure 2. The Prognostic value of REEP4. (**A**) Effect of receptor accessory protein 4 (REEP4) on disease-free survival of patients with lower-grade glioma (LGG). (**B**) Meta-analysis showing that elevated REEP4 expression is a risk prognostic factor for LGG (HR = 1.45, 95% CI: 1.08; 1.94). Abbreviations: HR: hazard ratio; CI: confidence interval. (**C**, **D**) Univariate and multivariate analysis based on TCGA RNA-seq database show that REEP4 is a risk factor for LGG prognosis. (**E**, **F**) Univariate and multivariate analysis based on CGGA microarray database show that REEP4 is a risk factor for LGG prognosis. (**G**, **H**) Univariate and multivariate analysis based on CGGA RNA-seq database show that REEP4 is a risk factor for LGG prognosis.

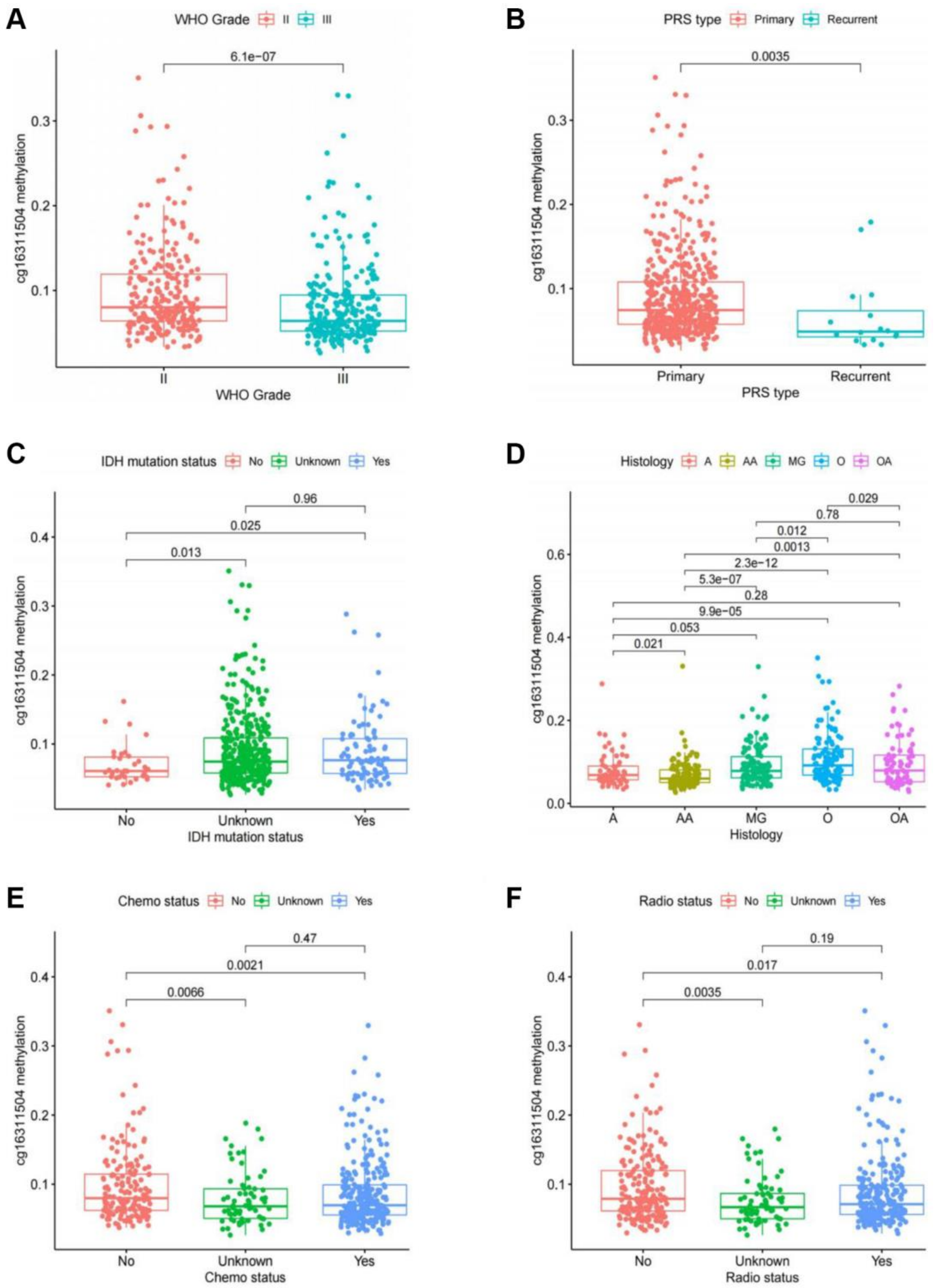

Supplementary Figure 3. Relationship between methylation site cg16311504 and the clinical characteristics of lower-grade glioma (LGG). (**A**) World Health Organization grade. (**B**) PRS type. (**C**) Isocitrate dehydrogenase mutation status. (**D**) Histological subtype. (**E**) Chemotherapy status. (**F**) Radiotherapy status.

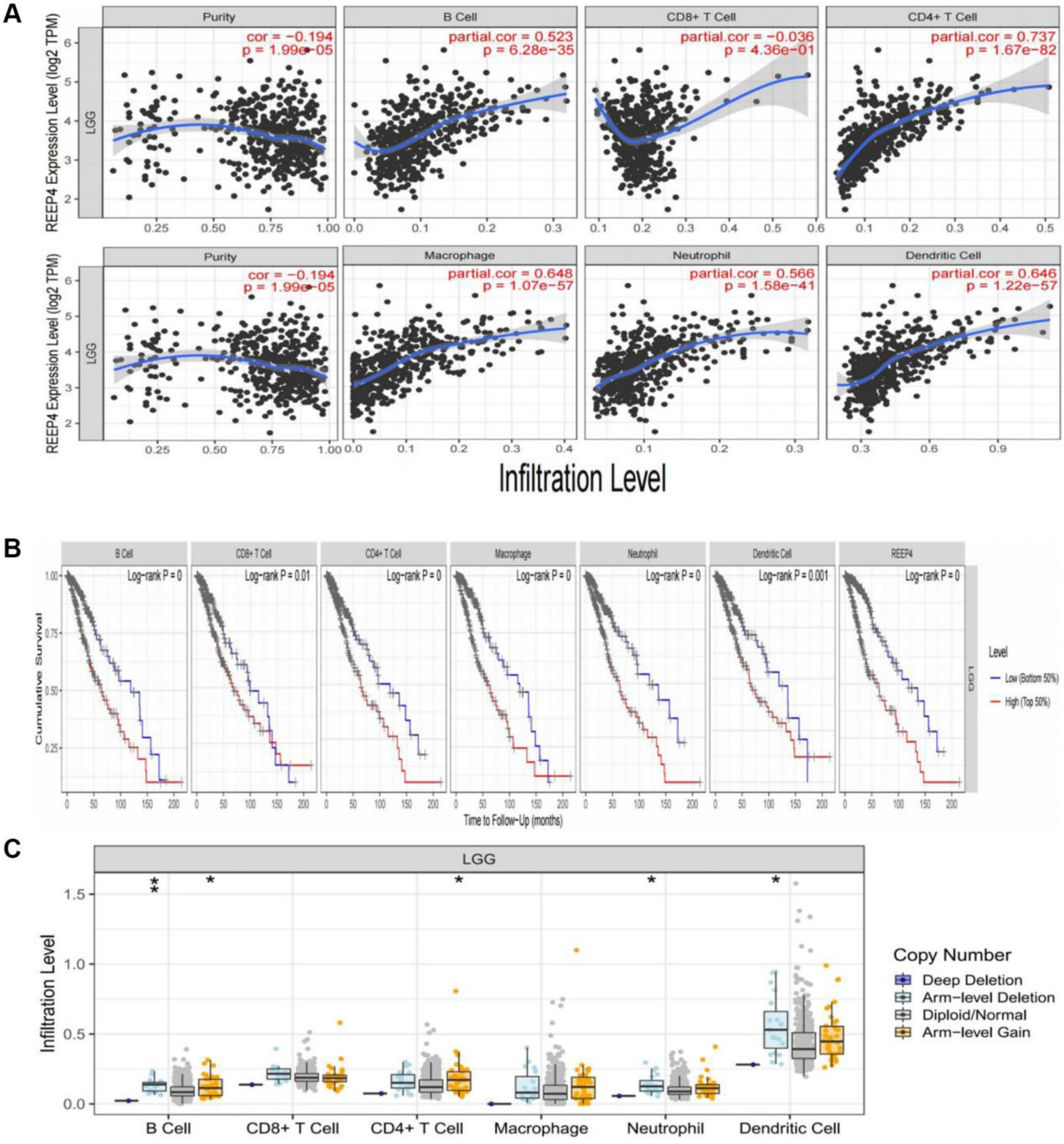

Supplementary Figure 4. The relationship between receptor accessory protein 4 (REEP4) expression and immune infiltration in lower-grade glioma (LGG). (**A**) REEP4 is negatively correlated with tumor purity and CD8+ T cells and positively correlated with B cells, CD4+ T cells, macrophages, neutrophils, and dendritic cells. (**B**) Six kinds of immune cells are negatively correlated with the overall survival of patients with LGG. (**C**) Relationship between the copy number variation of REEP4 and immune cell infiltration in LGG. $^{*}P < 0{\cdot}05$. $^{**}P < 0{\cdot}01$.
