# Supplementary material for: Potential value of expression of receptor accessory protein 4 for evaluating the prognosis of lower-grade glioma patients
Source: Aging (Albany NY). 2024 Mar 28;16(7):6188–211. doi: 10.18632/aging.205695 (PMC11042925; doi:10.18632/aging.205695)
Supplement: Supplementary Tables [file aging-16-205695-s002.pdf]

SUPPLEMENTARY TABLES

Supplementary Table 1. The detailed clinical features of LGG patients in TCGA RNA-seq.

Covariates	Type	Total	Percentages (%)
Age	≤41	260	51.69%
	>41	243	48.31%
Gender	Female	225	44.73%
	Male	278	55.27%
WHO Grade	II	243	48.31%
	III	260	51.69%
Radio status	No	187	37.18%
	Unknown	72	14.31%
	Yes	244	48.51%
Chemo status	No	167	33.20%
	Unknown	66	13.12%
	Yes	270	53.68%
PRS type	Primary	489	97.22%
	Recurrent	14	2.78%
IDH mutation status	No	34	6.76%
	Unknown	378	75.15%
	Yes	91	18.09%
expression	High	251	49.90%
	Low	252	50.10%
methylation	High	251	49.90%
	Low	252	50.10%

Supplementary Table 2. The detailed clinical features of LGG patients in CGGA microarray.

Covariates	Type	Total	Percentages (%)
PRS type	Primary	127	89.44%
	Recurrent	15	10.56%
WHO Grade	II	92	64.79%
	III	50	35.21%
Gender	Female	66	46.48%
	Male	76	53.52%
Age	≤41	83	58.45%
	>41	59	41.55%
Radio status	No	18	12.68%
	Yes	124	87.32%
Chemo status	No	79	55.63%
	Yes	63	44.37%
IDH mutation	No	48	33.80%
	Yes	94	66.20%
1p19q codeletion	No	31	21.83%
	Unknown	97	68.31%
	Yes	14	9.86%
MGMTp methylation	No	102	71.83%
	Yes	40	28.17%

Supplementary Table 3. The detailed clinical features of LGG patients in CGGA RNA-seq.

Covariates	Type	Total	Percentages (%)
PRS type	Primary	273	67.74%
	Recurrent	130	32.26%
WHO Grade	II	177	43.92%
	III	226	56.08%
Gender	Female	171	42.43%
	Male	232	57.57%
Age	≤41	222	55.09%
	>41	181	44.91%
Radio status	No	88	21.84%
	Yes	315	78.16%
Chemo status	No	134	33.25%
	Yes	269	66.75%
IDH mutation	No	100	24.81%
	Yes	303	75.19%
1p19q codeletion	No	280	69.48%
	Yes	123	30.52%
MGMTp methylation	No	165	40.94%
	Yes	238	59.06%
